# Regional Factors Related to Social Disconnection among Older Adults in South Korea: The Role of Subjective Health

**DOI:** 10.1093/geroni/igaf122.2787

**Published:** 2025-12-31

**Authors:** Jaeyoon Kim, Meejung Chin

**Affiliations:** Seoul National University, Seoul, Republic of Korea; Seoul National University, Seoul, Republic of Korea

## Abstract

Despite longstanding concerns about social isolation and loneliness among older adults, research on the macro-level factors (e.g., regional characteristics) influencing these issues remain limited. Using data from 6,045 older adults (aged 59–103) drawn from the ninth wave (2022) of the Korean Longitudinal Study of Aging and the Korean Statistical Information Service, we conducted multilevel modeling to analyze the relationships between regional factors and social disconnection (i.e., social isolation and loneliness) among older adults. Additionally, we examined whether subjective health status moderates these relationships. The results revealed that social isolation and loneliness were related to different regional factors. Older adults tended to be more isolated in regions with a lower proportion of single-person households aged 65 and older. Meanwhile, they were more likely to feel lonely in regions with a higher fiscal self-reliance rate and more leisure facilities per 1,000 older adults. Subjective health status amplified the relationship between fiscal self-reliance rate and both forms of social disconnection but reversed the relationship between proportion of social welfare spending and loneliness. Although higher fiscal autonomy was associated with lower social isolation across all health groups, it was related to greater loneliness, particularly among those in poorer health. In contrast, a higher proportion of welfare spending was linked to lower loneliness among those in poor health but to higher loneliness among those in good health. These findings underscore the need to consider both regional factors and the Person-Environment Fit (P-E Fit) framework in addressing social disconnection among older adults.

